# Comprehensive Review of the Ocular Toxicities Associated With Antibody-Drug Conjugates Used to Treat Gynecological Cancers

**DOI:** 10.7759/cureus.87453

**Published:** 2025-07-07

**Authors:** Jason Peng, Akhila Tetali, Amanda Malik, Rebeca Kelly

**Affiliations:** 1 Medical School, Cooper Medical School of Rowan University, Camden, USA; 2 Obstetrics and Gynecology, Cooper University Hospital, Camden, USA

**Keywords:** antibody drug conjugate, gynecological cancer, mirvetuximab soravtansine, ocular toxicity, tisotumab vedotin

## Abstract

This comprehensive literature review investigates the ocular toxicities associated with antibody-drug conjugates (ADCs) used in the treatment of gynecological cancers. Gynecological cancers, including uterine, ovarian, and cervical, pose a significant health burden with varying incidence and survival rates. Despite advancements in treatment modalities, survival rates remain moderate to relatively low for advanced or recurrent gynecologic cancers. ADCs, a targeted therapy utilizing antibody-antigen interactions, have emerged as a promising chemotherapeutic approach, delivering cytotoxic agents to cancer cells with high precision. Twenty-one papers were identified and analyzed to provide a comprehensive overview of the incidence, prevalence, underlying mechanisms, risk factors, and current management strategies for the ocular toxicities of ADCs. Currently, mirvetuximab soravtansine (MIRV) and tisotumab vedotin (TV) are Food and Drug Administration (FDA)-approved ADCs used in the treatment of gynecological cancers, demonstrating efficacy in clinical trials. However, ocular toxicities, particularly blurred vision, keratopathy, and conjunctivitis, are commonly reported in patients receiving these treatments. When combined with bevacizumab and carboplatin, these drugs are associated with increased ocular adverse events. Further research is warranted to better understand the long-term effects and mechanisms underlying ocular toxicities induced by ADCs. In addition, a standardized reporting system is recommended to facilitate this process. We therefore aim to provide a thorough understanding of ocular toxicities in ADCs, with the objective of optimizing patient care within the field of gynecological oncology and contributing to the improvement of patient outcomes.

## Introduction and background

Gynecological cancers include cancers of the vagina, vulva, cervix, uterus, ovaries, and fallopian tubes [1]. From 2012 to 2016, 94,000 women were diagnosed with gynecologic cancers, the most common being uterine cancer (26.82 cases per 100,000) and least common being vaginal cancer (9.60 per 100,000) [2]. In 2021, the American Cancer Society estimated 116,760 new cases of gynecological cancers and 34,080 resulting deaths in the United States alone [1]. Despite recent advancements in gynecologic cancer treatment, survival rates remain moderate, with a five-year survival rate of 50.8% for ovarian cancer, 67.2% for cervical cancer, and 81% for uterine cancer in all stages [3]. The primary treatment options include surgery with a combination of platinum-based chemotherapy and/or radiation [3]. Platinum-based chemotherapy is often used concurrently with paclitaxel or one of the following drugs to improve progression-free survival in advanced disease: (1) vascular endothelial growth factor (VEGF) inhibitors (i.e. bevacizumab or lenvatinib), (2) programmed cell death (PD-1) inhibitors (i.e. pembrolizumab) with or without VEGF inhibitors, or (3) novel poly(adenosine diphosphate (ADP)-ribose) polymerase inhibitors [3]. In metastatic or recurrent cases, which have low cure rates, the need for more efficient therapies has led to the investigation of antibody-drug conjugates (ADCs).

ADCs are targeted therapies that utilize antibody-antigen interactions to deliver drugs and cytotoxic agents to tumor cells and tumor microenvironments with high precision, maximizing clinical efficacy and minimizing toxicity [3]. They also activate the immune system, causing immunogenic cell death [3]. They consist of an antibody, cytotoxic payload, and linker [4]. Table 1 shows the vital role of each component and the special considerations that allow for optimal functioning [3,4].

**Table 1:** Function and Considerations of the Components of Antibody-Drug Conjugates This table was created by the authors based on information adapted from sources published under a Creative Commons Attribution License (CC BY-NC-ND 4.0) [3].

Component	Function	Consideration
Antibody	Binds specific antigens on the tumor cell [3]	Must bind the target antigen with high affinity and be effectively internalized by receptor-mediated endocytosis, target antigens with high expression on tumor cells and low to no expression in healthy tissue, and have limited immunogenic effects [3]
Cytotoxic payload	Acts as an inhibitor of certain cell cycle components (i.e. microtubules, nicotinamide phosphoribosyltransferase, etc.) or as DNA-damaging agents [3]	Must be high potency as limited tumor penetration is anticipated [3]
Linker	Connects the antibody and payload [3]	Must be highly stable in circulation, not releasing the payload before delivery to the target but efficiently releasing it inside the tumor cell [3]

ADCs work through the following series of events: The antibody recognizes the cancer cell’s tumor antigen, be internalized by receptor-mediated endocytosis, the ADC travels to the cytoplasm and is degraded by the endolysosomal component, the payload is released into the cytoplasm, and the payload inhibits the cell cycle component or DNA, inducing cell death [5]. There are currently only two approved ADCs for use against gynecological cancers. The first, tisotumab vedotin (TV), was approved by the Food and Drug Administration (FDA) in September 2021 for recurrent or metastatic cervical cancer with disease progression on or after chemotherapy [6]. TV targets tissue factor using the payload monomethyl auristatin E [4]. Mirvetuximab soravtansine (MIRV) was approved by the FDA in November 2022 for adult patients with folate receptor alpha (FRa)-positive, platinum-resistant epithelial cancer of ovarian, fallopian tube, or primary peritoneal origin, who have received one to three prior systemic treatment regimens [6]. MIRV targets FRa using the maytansinoid payload ravtansine, a tubulin-targeting agent [4]. Although ADCs in general are designed to have a wide therapeutic index, they still have many adverse effects. 

The most common all-grade adverse events for ADCs in general include lymphopenia (53.0%; 95% confidence interval (CI), 48.7%-57.3%), nausea (44.1%; 95% CI, 43.2%-44.9%), neutropenia (43.7%; 95% CI, 42.6%-44.9%), blurred vision (40.5%; 95% CI, 37.4%-43.6%), and peripheral neuropathy (39.6%; 95% CI, 38.2%-41.1%) [7]. For TV and MIRV, as well as for belantamab mafodotin, an ADC used to treat multiple myeloma, ocular toxicity is a common adverse effect [3,8]. Of note, one review looked at 22 primary studies and found that the most commonly cited adverse effects included blurred vision (10/22 studies), dry eyes (7/22 studies), and corneal microcysts (5/22 studies), corneal deposits and inclusions (4/22 studies), and conjunctivitis/keratoconjunctivitis (3/22 studies) [3,8]. Although the mechanism and pathogenesis of these toxicities are not well understood, past studies have hypothesized many explanations [8]. These include the auristatin or maytansinoid payloads damaging the eye’s different components due to the eye’s inherently vast blood supply, the presence of rapidly dividing cell subpopulations, and the abundance of cell surface receptors [8]. Current management includes the use of corticosteroid and vasoconstrictor eye drops, as well as dose interruptions and modifications, all of which have demonstrated good responses [4].

This comprehensive review will evaluate the current literature to describe our understanding of ocular toxicities caused by ADCs used to treat gynecological cancers. The most common ocular toxicities, their incidence and prevalence, likely underlying mechanisms, risk factors, current management strategies, and gaps in knowledge will all be addressed. Given the rapidly evolving field of ADCs, this review focuses on the currently available knowledge of ADC-induced ocular toxicities and considers all ADCs that cause ocular toxicity (even those not used to treat gynecological cancers) in order to provide a more comprehensive evaluation of the mechanism behind such toxicity.

## Review

Materials and methods

A literature search was conducted on July 2, 2023, using the following electronic databases: PubMed (https://pubmed.ncbi.nlm.nih.gov/, accessed July 2, 2023) and Ovid MEDLINE (accessed July 2, 2023). The purpose of this review was to evaluate current literature on ADCs, primarily those used to treat gynecological cancers, and their ocular toxicities. This study explores ocular symptoms, their incidence, mechanisms of action, risk factors, management strategies, and knowledge gaps.

The search strategy divided terms into grouped categories. PubMed was first searched to assess whether Medical Subject Heading (MeSH) terms returned relevant articles and identify keyword equivalents to improve search sensitivity. Search terms included “Gynecology,” “Chemotherapy,” “antibody drug conjugate,” “ocular toxicity,” “Tisotumab vedotin,” “Mirvetuximab soravtansine,” “Belantamab mafodotin,” “Trastuzumab deruxtecan,” “Eye,” “Ophthalmologic,” “Vision,” “Keratitis,” “Cornea,” “Corneal microcysts,” “Conjunctivitis,” “Dry eye,” “Uveitis,” “Cataract,” “Neuropathy,” “Retina,” and “Blindness.” Paired search terms included "ocular toxicity, Tisotumab vedotin", "ocular toxicity, Mirvetuximab soravtansine", "ocular toxicity, Belantamab mafodotin", "ocular toxicity, Trastuzumab deruxtecan", and "antibody drug conjugate, eye", among others.

The initial search yielded 17,638 articles, reduced to 5,188 after removing duplicates. Approximately 10-20 articles from each paired search term were reviewed for relevance. Terms such as "gynecology, chemotherapy", "gynecology, antibody drug conjugate", and "antibody drug conjugate, neuropathy" were excluded for deviating from the topic. Terms like "ocular toxicity, Trastuzumab deruxtecan", "antibody drug conjugate, corneal microcysts", and "cataract" were removed due to lack of results or overlap with more comprehensive groupings.

**Figure 1:**
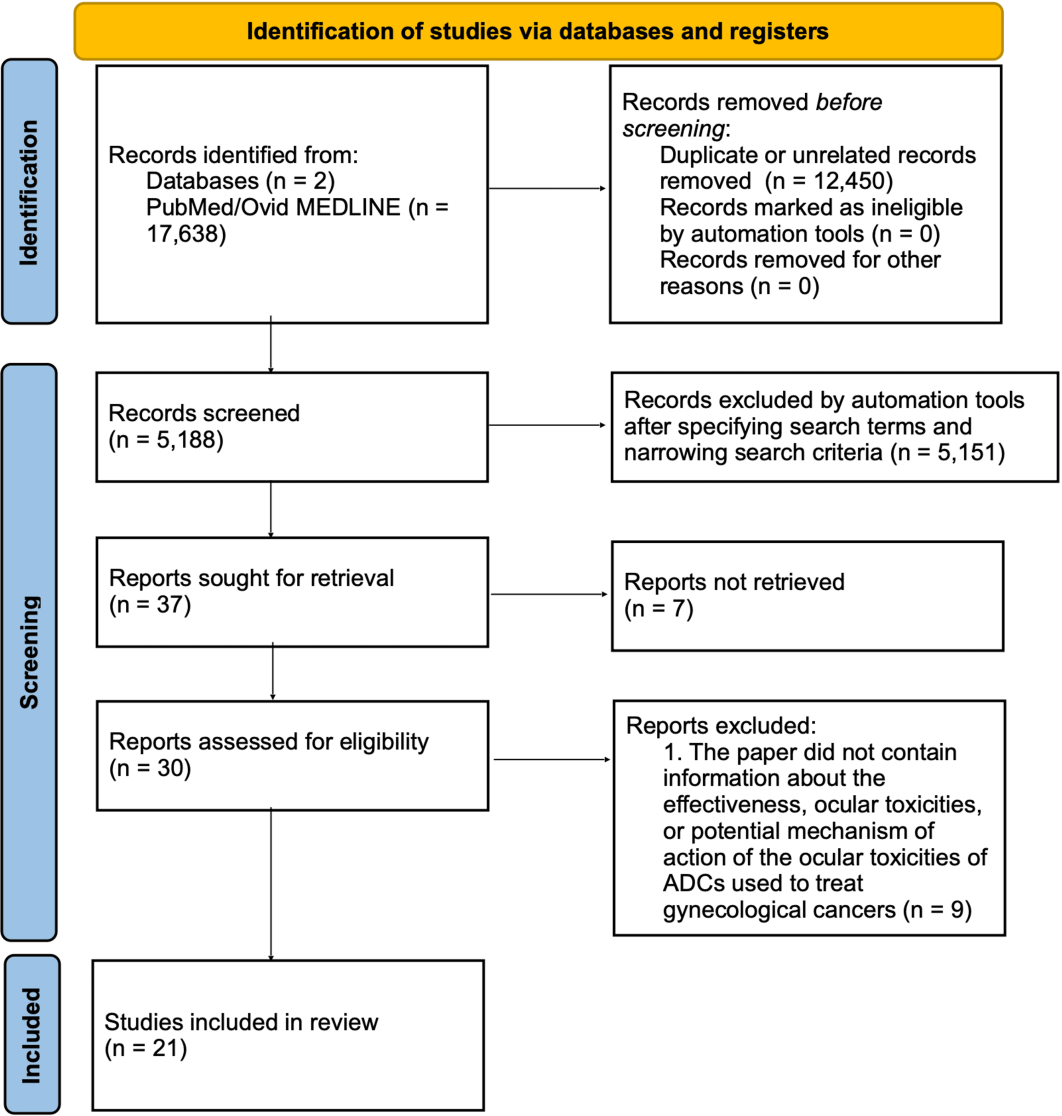
Preferred Reporting Items for Systematic Reviews and Meta-Analyses (PRISMA) flow diagram of article selection process ADCs=Antibody-drug conjugates

This refinement left 164 relevant papers. The final search query, combining grouped terms, was encoded in PubMed and Ovid MEDLINE as (("Ocular toxicity" (All Fields) OR "Eye"[All Fields] OR "Ophthalmologic" (All Fields) OR "Vision" (All Fields)) AND ("Tisotumab vedotin" (All Fields) OR "Mirvetuximab soravtansine" (All Fields) OR "Belantamab mafodotin" (All Fields) OR "Antibody drug conjugate" (All Fields)). This search returned 101 articles. After applying filters - (1) publication dates from January 1, 2010 to July 1, 2023, (2) humans only, (3) English only, and (4) adult age group (19+) only - 37 articles remained. Of these, seven were excluded due to lack of access, and nine due to irrelevance (not addressing ocular toxicities or mechanisms of ADCs for gynecological cancers), leaving 21 articles for analysis. The reference lists of the included papers were also screened, but no additional articles were removed. Figure 1 shows the Preferred Reporting Items for Systematic Reviews and Meta-Analyses (PRISMA) flow diagram of the article selection process.

Extracted information included the following: drug of focus, study type, location, population, limitations, and statistical data such as objective response rate; complete and partial response rates; progression-free survival; median duration of response; and median follow-up. All three authors independently evaluated the quality and risk of bias for each study using a customized checklist adapted from the National Institutes of Health (NIH) Study Quality Assessment Tools. Key criteria included: clarity of objectives, patient selection, outcome consistency, and adequacy of follow-up. Discrepancies were resolved by consensus, and studies were categorized as low, moderate, or high risk of bias to guide synthesis.

Results

Twenty-one papers - including 14 clinical trials, two cohort studies, one comparative study, one cross-sectional study, one observational study, one case study, and one review - on ADC-associated ocular toxicities in gynecological cancer treatment were analyzed. Nine papers focused on MIRV, six on belantamab mafodotin, three on TV, and the remaining on other ADCs. This analysis incorporated data from 5,181 patients across six countries. Key study characteristics and limitations are presented in Table 2.

**Table 2:** List of the 21 Studies Analyzed with their Key Characteristics and Limitations RECIST: Response Evaluation Criteria in Solid Tumors; ROBINS: Risk of Bias in Non-Randomised Studies; AE: adverse effects; ADCs: antibody-drug conjugates

Author	Year	Country	Study Type	Population	Cancers Cited to be Treated	Drug of Focus	Limitation
Moore et al. [9]	2016	United States	Clinical Trial	46	Platinum-resistant ovarian, fallopian tube, and primary peritoneal cancer	Mirvetuximab soravansine	The study population was relatively small, with only 46 patients enrolled. The study focused on patients with platinum-resistant ovarian cancer and may not fully represent the broader population of gynecological cancer patients. The study did not include a control group for comparison. The study did not evaluate long-term outcomes, such as overall survival. The population evaluated was predominantly white, limiting generalizability to other racial or ethnic groups.
Moore et al. [10]	2017	United States	Clinical Trial	44	Ovarian cancer	Mirvetuximab soravtansine	Small Sample Size: The study included a relatively small sample size of 44 patients. A larger sample size would have provided more robust data and allowed for a better assessment of ocular toxicities associated with ADCs used to treat gynecological cancers. Heterogeneous Tumor Types: The study included both gynecological and non-gynecologic malignancies. Although the majority of patients had gynecological cancers, the inclusion of non-gynecologic malignancies might introduce heterogeneity in the study population and could potentially affect the interpretation of the ocular toxicities. Single-Arm Study Design: The study utilized a single-arm design without a comparator group. This limits the ability to directly compare ocular toxicities between different ADCs or treatment regimens. Limited Follow-up Period: The study may have had a relatively short follow-up period, which could impact the detection and reporting of ocular toxicities that occur after prolonged exposure to ADCs.
O'Malley et al. [11]	2020	United States	Clinical Trial	66	Platinum-resistant ovarian cancer	Mirvetuximab soravtansin w bevacizumab	The review is based on a systematic analysis of the existing literature, which may have variations in study design, patient populations, and reporting of ocular toxicities associated with ADCs. The sample size of the included studies varied, and some studies may have had a small number of participants, limiting the generalizability of the findings. The review may not include the most recent studies published after the literature cutoff date. The study focused on patients with platinum-resistant gynecological cancers receiving specific combination treatment, which may limit the generalizability of the findings to other patient populations or treatment regimens.
Gilbert et al. [12]	2023	Canada	Comparative Study	94	Platinum-resistant ovarian cancer	Mirvetuximab soravtansine w bevacizumab	The study had a relatively small sample size, with only 94 patients included in the analysis. The study focused on patients with platinum-resistant gynecological cancers receiving specific combination treatment, which may limit the generalizability of the findings to other patient populations or treatment regimens. The follow-up period for assessing long-term outcomes was limited, and longer-term data on overall survival were not reported. The study did not include a control group or comparative analysis with other treatment options, which limits the ability to make direct comparisons or assess the relative efficacy and safety of the combination treatment.
Coleman et al. [13]	2021	United States	Clinical Trial	102	Previously treated recurrent or metastatic cervical cancer	Tisotumab vedotin	The sample size of the included studies was relatively small, with a total of 101 patients receiving ADC treatment for gynecological cancers. The studies included in the review might have heterogeneity in terms of study design, patient populations, and reporting of ocular toxicities. Some studies might have had a short follow-up duration, limiting the assessment of long-term ocular toxicities.
de Bono et al. [14]	2019	United Kingdom	Clinical Trial	147	Relapsed, advanced, or metastatic cancer of the ovary, cervix, endometrium, bladder, prostate, oesophagus, squamous cell carcinoma of the head and neck or non-small-cell lung cancer	Tisotumab vedotin	The review is based on the current literature available up to the cutoff date and may not capture recent developments in the field. The included studies may have variations in study design, patient populations, and outcome measures, which could introduce heterogeneity. The sample size of the reviewed studies may be limited, potentially affecting the generalizability of the findings.
Matulonis et al. [15]	2019	United States	Clinical Trial	40	Platinum-resistant ovarian cancer	Mirvetuximab soravtansine	The study relies on data from clinical trials and preclinical animal models, which may not fully represent the real-world population or clinical settings. The sample size of the expansion cohort for evaluating primary prophylactic corticosteroid eye drop use is relatively small. The study focuses specifically on mirvetuximab soravtansine and its ocular toxicities, potentially limiting generalizability to other ADCs. The analysis of ocular adverse event profiles is based on descriptive statistics, and no formal statistical hypothesis testing is mentioned.
Kunkler et al. [16]	2019	United States	Observational Study	3537	Ovarian cancer	Mirvetuximab soravtansine	The study was a retrospective chart review conducted at a single academic Eye Institute and affiliated Comprehensive Cancer Center, which may limit the generalizability of the findings. The sample size of the study was relatively small, with 31 patients identified with ocular side effects. The study relied on chart documentation, which may be subject to variations in recording and reporting. The duration of follow-up and long-term outcomes of the patients were not discussed in detail. The study did not compare the incidence and prevalence of ocular toxicities between different types of cancers or different ADCs.
Martin et al. [17]	2017	United States	Clinical Trial	27	Relapsed epithelial ovarian cancer patients	Mirvetuximab soravtansine	Small sample size
Moore et al. [18]	2018	United States	Clinical Trial	18	Platinum-sensitive ovarian cancer	Mirvetuximab soravtansine w carboplatin	Limited Sample Size: The review included a relatively small sample size of only 18 patients. This small sample size may affect the generalizability of the findings to a broader population of patients with gynecological cancers. Time Constraints: The review focused on studies published between December 2015 and November 2016. This time frame may not capture the most recent developments and advancements in the field of ADCs and ocular toxicities. Heterogeneity of Studies: The included studies may vary in terms of study design, patient populations, and ADC agents used. This heterogeneity may introduce potential biases and limit the comparability of the findings across studies.
Luu et al. [19]	2022	United States	Review	N/A	Recurrent or metastatic cervical cancer	Tisotumab vedotin	Sample Size: The review included a comprehensive analysis of the current literature on ocular toxicities associated with ADCs used to treat gynecological cancers. However, the sample size of the studies varied, and some studies may have had small sample sizes, limiting the generalizability of the findings. Publication Bias: There is a possibility of publication bias, as studies reporting positive outcomes or significant findings are more likely to be published. This bias may affect the overall representation of ocular toxicities associated with ADCs. Heterogeneity of Studies: The included studies may have used different methodologies, patient populations, and treatment regimens, which could introduce heterogeneity in the data. This heterogeneity may affect the ability to draw definitive conclusions or make direct comparisons across studies. Knowledge Cutoff: The review's findings are based on the literature available up until the knowledge cutoff date of September 2021. Newer studies or developments in the field may not be included, and the review may not capture the most recent understanding of ocular toxicities associated with ADCs. Reporting Bias: The review's findings are reliant on the data reported in the included studies. Incomplete or inadequate reporting of ocular toxicities in the literature may limit the comprehensiveness of the review's findings.
Banerji et al. [20]	2019	United Kingdom	Clinical Trial	185	HER2-positive breast, gastric, urothelial, or endometrial cancer	Trastuzumab duocarmazine	The study period only covers data between October 2014 and April 2018, limiting the analysis to a specific timeframe. The sample size is relatively small, with 39 patients in the dose-escalation phase and 146 patients in the dose-expansion phase, which may affect the generalizability of the findings. The follow-up period was relatively short, with a median follow-up of 5.0 months, which may limit the assessment of long-term ocular toxicities.
Shapiro et al. [21]	2017	United States	Clinical Trial	26	Lung and breast cancer	PF-06263507	The small sample size: With only 26 patients receiving treatment, the study may have limited statistical power to detect less common adverse events or to draw definitive conclusions about the drug's efficacy. Lack of a control group: The study did not compare the treatment with PF-06263507 to a placebo or another standard treatment, making it difficult to determine the drug's effectiveness. Short duration of the study: The treatment period ranged from August 2013 to March 2015, which may not have been sufficient to assess long-term outcomes or potential late-onset adverse events.
Liu et al. [22]	2021	United States	Clinical Trial	65	Platinum-resistant ovarian cancer	DMUC4064A	Small sample size
Lonial et al. [23]	2020	United States	Clinical Trial	196	Relapsed or refractory multiple myeloma	Belantamab mafodotin	The studies included in the review might have heterogeneity in terms of study design, patient populations, and reporting of ocular toxicities.
Baines et al. [24]	2022	United States	Cohort Study (Multicenter)	251	Relapsed or Refractory Multiple Myeloma	Belantamab mafodotin	The review focused specifically on ocular toxicities associated with ADCs used to treat gynecological cancers. Therefore, the findings may not be directly applicable to ADCs used to treat other types of cancer or non-gynecological cancers. The review was based on the available literature up until the knowledge cutoff date of September 2021. Newer studies or information published after this date may not be included in the review.
Richardson et al. [25]	2020	United States	Clinical Trial	31	Relapsed/refractory multiple myeloma	Belantamab mafodotin	Small sample size and the study was conducted at a limited number of sites in the United States and Australia
Shragai et al. [26]	2023	Israel	Cohort Study	106	Relapsed/refractory multiple myeloma	Belantamab mafodotin	The study has several limitations. It was retrospective in nature and relied on data extracted from electronic medical charts, which may introduce bias and incomplete information. The sample size was relatively small, and the study included a heterogeneous population of relapsed/refractory multiple myeloma patients. The study also did not compare the outcomes with a control group or other treatment modalities, limiting the ability to draw direct comparisons.
Marquant et al. [27]	2021	France	Case Study	1	Multiple myeloma	Belantamab mafodotin	The study is based on a single patient case, which limits generalizability to a broader population. The sample size is small, which may affect the robustness of the findings. The study lacks a control group, making it difficult to assess the causal relationship between belamaf treatment and ocular toxicities. The duration of follow-up is relatively short, which may not capture long-term ocular effects. The study lacks a comparison of ocular toxicities with other ADCs used to treat gynecological cancers, which could provide more comprehensive insights into the ocular effects of ADCs. The study does not address the ocular toxicities of ADCs used to treat gynecological cancers, as the focus is solely on belamaf in multiple myeloma patients.
Ferron-Brady et al. [28]	2021	United States	Clinical Trial	194	Relapsed/Refractory Multiple Myeloma	Belantamab mafodotin	Study Design and Population: The limitations of the original studies, such as their design, patient population, and potential confounding factors, could impact the interpretation of the findings.
Corbelli et al. [29]	2019	Italy	Cross-sectional Study	5	Advanced epithelial ovarian cancer	Mirvetuximab soravtansine	The sample size is small, consisting of only five female patients. The study period covers only 5 months, limiting the long-term evaluation of ocular toxicities. The study is retrospective and lacks a control group for comparison. The study focuses solely on mirvetuximab soravtansine and does not provide a comprehensive evaluation of ocular toxicities associated with other antibody-drug conjugates.

Discussion

Summary of Main Results

This analysis examines each drug individually and by category: FDA-approved for gynecologic cancers, drugs with potential antitumor effects, and drugs FDA-approved for other diseases. Safety and efficacy are not discussed in this review; ocular symptoms were reviewed for all. A detailed risk of bias assessment was conducted for all 21 studies using the Cochrane and ROBINS-I (Risk of Bias in Non-Randomised Studies) tools, evaluating domains such as randomization, intervention deviations, missing data, outcome measurement, and selective reporting. The results are summarized in Table 3. Of the studies, four were classified as high risk, 12 as moderate risk, and five as low risk of bias. High-risk studies were used less frequently in drawing conclusions, increasing the validity of interpretations regarding ADC ocular toxicities. Further, a list of the medications reviewed in this study, their associated cancer indications, reported ocular complications, and relevant incidence rates can be found in Table 4.

**Table 3:** Risk of Bias Assessment for each of the 21 Included Studies ROBINS: Risk of Bias in Non-Randomised Studies.

Author	Year	Study Type	Population	Drug of Focus	Design	Risk of Bias Tool	Overall Risk of Bias	Notes
Moore et al. [9]	2016	Clinical Trial	46	Mirvetuximab soravansine	Phase I, single-arm expansion	ROBINS-I	Moderate	No control arm; confounding risks; open-label design may introduce selection bias; small sample size; RECIST 1.1 applied; all outcomes reported.
Moore et al. [10]	2017	Clinical Trial	44	Mirvetuximab soravtansine	Phase I, dose-escalation (3+3)	ROBINS-I	Moderate	No comparator; single-patient cohorts risk selection bias; intervention/dosing clear; consistent with standard phase I outcome reporting.
O’Malley et al. [11]	2020	Clinical Trial	66	Mirvetuximab soravtansin w bevacizumab	Phase Ib, single-arm combo	ROBINS-I	Moderate	Bevacizumab confounds treatment effect; open-label risk; missing data not a concern; RECIST 1.1 and AE reporting robust.
Gilbert et al. [12]	2023	Comparative Study	94	Mirvetuximab soravtansine w bevacizumab	Single-arm interventional	ROBINS-I	Moderate	No control arm; eligibility rigorous; no major attrition or deviations; RECIST and AE grading used; transparent reporting.
Coleman et al. [13]	2021	Clinical Trial	102	Tisotumab vedotin	Open-label, single-arm, phase 2 study	Cochrane Risk of Bias Tool	High	Open-label, single-arm design; lack of randomization and control group increases risk of bias.
de Bono et al. [14]	2019	Clinical Trial	147	Tisotumab vedotin	Open-label, dose-escalation and dose-expansion, phase 1–2 study	Cochrane Risk of Bias Tool	High	Open-label, dose-escalation design; lack of randomization and control group increases risk of bias.
Matulonis et al. [15]	2019	Clinical Trial	40	Mirvetuximab soravtansine	Phase I non-randomized expansion cohort with comparison to historical or concurrent groups without prophylaxis	ROBINS-I	Moderate	Confounding risk due to lack of randomization and unclear matching of groups. Blinding not reported for outcome measurement, but other domains had low risk.
Kunkler et al. [16]	2019	Observational Study	3537	Mirvetuximab soravtansine	Retrospective case series	ROBINS-I	High	High confounding and selection bias. Incomplete outcome data due to loss to follow-up. No standardized outcome measurement.
Martin et al. [17]	2017	Clinical Trial	27	Mirvetuximab soravtansine	Phase I study with exploratory biomarker-outcome association	ROBINS-I	Moderate	Moderate confounding due to lack of control group. Some missing data and no blinding of outcome assessors.
Moore et al. [18]	2018	Clinical Trial	18	Mirvetuximab soravtansine w carboplatin	Phase Ib dose-escalation non-randomized trial	ROBINS-I	Moderate	No control group, confounding possible. Clear participant selection and well-defined interventions. All patients followed and complete outcome reporting.
Luu et al. [19]	2022	Review	N/A	Tisotumab vedotin	Narrative review article	ROBINS-I	High	Not a clinical study. Confounding not applicable. Selective reporting and possible bias due to narrative review structure.
Banerji et al. [20]	2019	Clinical Trial	185	Trastuzumab duocarmazine	Phase I dose-escalation and expansion	ROBINS-I	Moderate	Key concerns: Lack of randomization, investigator-assessed outcomes, heterogeneity in prior treatments.
Shapiro et al. [21]	2017	Clinical Trial	26	PF-06263507	Anti-5T4 ADC	ROBINS-I	Moderate	Key concerns: No biomarker enrichment (5T4), no objective responses, pretreated patients with advanced tumors.
Liu et al. [22]	2021	Clinical Trial	65	DMUC4064A	Anti-MUC16 ADC	ROBINS-I	Moderate	Key concerns: Investigator-based response assessment, variability in prior treatments in ovarian cancer cases.
Lonial et al. [23]	2020	Clinical Trial	196	Belantamab mafodotin	Randomized controlled trial, open-label	Cochrane Risk of Bias Tool	Low	Open-label design introduces potential bias in outcome assessment. Intention-to-treat analysis minimizes missing data bias.
Baines et al. [24]	2022	Cohort Study (Multicenter)	251	Belantamab mafodotin	Secondary analysis of Phase II trial, real-world context	ROBINS-I	Low	No clear control group, but robust data review with no evidence of selective reporting or intervention issues.
Richardson et al. [25]	2020	Clinical Trial	31	Belantamab mafodotin	Cohort analysis of DREAMM-2 trial	Cochrane Risk of Bias Tool	Low	Cohort-specific analysis may introduce bias from treatment differences. Primary outcome assessment by independent review minimizes bias.
Shragai et al. [26]	2023	Cohort Study	106	Belantamab mafodotin	Retrospective, multicenter study	ROBINS-I	Moderate	Retrospective design introduces confounding factors; missing data and participant selection issues are notable concerns.
Marquant et al. [27]	2021	Case Study	1	Belantamab mafodotin	Case report, single patient	ROBINS-I	Low	Clear focus on a single patient with minimal confounding. Data completeness and reliable outcome measurement reduce bias.
Ferron-Brady et al. [28]	2021	Clinical Trial	194	Belantamab mafodotin	Secondary analysis of DREAMM-2 and DREAMM-1 trial data	Cochrane Risk of Bias Tool	Low	Analysis of existing data may have some limitations due to lack of new interventions or randomization, but uses validated models and established clinical measures.
Corbelli et al. [29]	2019	Cross-sectional Study	5	Mirvetuximab soravtansine	Case series	ROBINS-I	Moderate	Small sample size (five patients); no control group but detailed assessment of clinical outcomes and symptoms.

**Table 4:** Medications reviewed in this study with their associated cancer indications, reported ocular complications, and relevant incidence rates MIRV: Mirvetuximab soravtansine.

Drug	Cancer Indication	Reported Ocular Complications	Incidence/Values
Mirvetuximab soravtansine [9,10,15,16,17,29]	Platinum-resistant ovarian cancer	Blurred vision, keratopathy, increased lacrimation, dry eye, foreign-body sensation, photophobia, ocular pain, corneal microcysts, corneal flattening	Blurred vision: 41.33%; Keratopathy: 10.20%; Lacrimation: 2.55%; Dry eye: 2.04%; Others: ~2% or lower
MIRV+Bevacizumab [11,12]	FRα-positive, platinum-resistant ovarian cancer	Blurred vision, dry eye, keratopathy	Blurred vision: 54.38%; Dry eye: 11.25%; Keratopathy: 10.00%
MIRV+Carboplatin [18]	Platinum-resistant ovarian cancer	Blurred vision, keratopathy	Blurred vision: 61.11%; Keratopathy: 22.22%
Tisotumab vedotin [13,14,19]	Recurrent/metastatic cervical cancer	Conjunctivitis, dry eye, ulcerative keratitis	Conjunctivitis: 35.74%; Dry eye: 22.09%; Ulcerative keratitis: 0.80%
Trastuzumab duocarmazine [20]	Potential in gynecological cancers (HER2+)	Conjunctivitis, dry eye, lacrimation, keratitis, blurred vision, corneal toxicity, retinal hemorrhage	Conjunctivitis and dry eye: 30.82%; Lacrimation: 19.86%; Keratitis: 19.18%; Blurred vision: 10.96%; Corneal and retinal: 0.68%
PF-06263507 [21]	Potential in ovarian and various cancers	Photophobia, dry eye, ocular pain, blurred vision, conjunctivitis, lacrimation, vitreous floaters	Photophobia: 26.92%; Dry eye: 23.08%; Ocular pain: 15.38%; Blurred vision: 11.54%; Others: 7.69% each
DMUC4064A [22]	Anti-MUC16 ADC for epithelial ovarian cancer	Blurred vision, dry eye, keratitis	Blurred vision: 35.38%; Dry eye: 16.92%; Keratitis: 13.85%
Belantamab mafodotin [23-28]	Multiple myeloma (associated risk for gynecologic cancers)	Keratopathy, blurred vision	Keratopathy: up to 75% (40.5% grade ≥3); Blurred vision: up to 36.8% (8% grade ≥3)

Results in the context of published literature

Drugs FDA Approved to Treat Gynecological Cancers

*Mirvetuximab soravtansine, monotherapy*: MIRV (IMGN853) combines a humanized anti-FRα monoclonal antibody and DM4 [4,7,10]. Six studies with 196 patients used the same MIRV dose. Ocular toxicities included blurred vision (81 patients, 41.33%), keratopathy (20 patients, 10.20%), increased lacrimation (five patients, 2.55%), dry eye (four patients, 2.04%), foreign-body sensation (four patients, 2.04%), photophobia (four patients, 2.04%), ocular pain (two patients, 1.02%), corneal microcysts (one patient, 0.51%), and corneal flattening (one patient, 0.51%) [9,10,15,16,17,29]. All symptoms were grade 1-2 on the Kinetic Visual Acuity (KVA) Scale. Blurred vision and keratopathy are common, but other symptoms need more research to confirm an association with MIRV. Ocular toxicities are detected using slit-lamp, anterior segment infrared (AS-IR), and anterior segment optical coherence tomography (AS-OCT) [9]. Management includes dose modification, lubricating and corticosteroid drops, which are effective [9]. A 40-patient study showed blurred vision decreased to 40% with corticosteroid prophylaxis [15]. Contact lenses should be avoided [9]. FRα expression in retinal tissue suggests retinal binding causes toxicity [15]; FRα is absent in cornea and non-retinal tissues, but rabbit studies confirmed corneal lesions with MIRV [15].

*Mirvetuximab soravtansine with bevacizumab:** ***Bevacizumab improves MIRV efficacy in FRα-positive, platinum-resistant ovarian cancers [11]. In 160 patients, ocular toxicities included blurred vision (87 patients, 54.38%), dry eye (18 patients, 11.25%), and keratopathy (16 patients, 10.00%) [11]. Bevacizumab may increase toxicity severity (Grade 3), but more research is needed.

*Mirvetuximab soravtansine with carboplatin*:** **Carboplatin was tested with MIRV in 18 patients. Ocular toxicities included blurred vision (11 patients, 61.11%) and keratopathy (four patients, 22.22%) [18]. This combination may raise toxicity risk, needing more study.

*Tisotumab vedotin*:** **TV targets tissue factor in solid tumors [14]. In 249 patients, ocular toxicities included conjunctivitis (89 patients, 35.74%), dry eye (55 patients, 22.09%), and ulcerative keratitis (two patients, 0.80%) [13,14]. Toxicity risk increases with dose, treatment duration, and concurrent drugs [13,14,19]. Early ophthalmologic care is crucial [13,14,19]; toxicities are generally reversible with dose changes or discontinuation [13,14,19]. Mechanisms likely involve off-target binding on ocular tissues [13,14,19]. Long-term effects are unclear; future research should address mechanisms and management strategies.

Drugs with Potential Antitumor Effects Against Gynecological Cancers

*Trastuzumab duocarmazine*:** **Trastuzumab duocarmazine is an ADC composed of trastuzumab, a recombinant humanized anti-HER2 monoclonal antibody, linked to duocarmycin, a prodrug with antineoplastic activity [20]. Initially approved for HER2-positive breast cancers, it may also show antitumor effects in gynecological cancers. Ocular toxicities were reported in 147 patients: conjunctivitis (30.82%; four grade 3), dry eye (30.82%; one grade 3), increased lacrimation (19.86%; all grades 1-2), keratitis (19.18%; three grade 3), blurred vision (10.96%; one grade 3), corneal toxicity (0.68%; one grade 3), and retinal hemorrhage (0.68%; one grade 3) [20]. Conjunctivitis and dry eye were most common; tearing, keratitis, and blurred vision occurred less frequently. Further research is needed to explore the mechanisms and compare with other drugs in Section 1.

*PF-06263507*:** **PF-06263507 is an ADC that inhibits tubulin polymerization and has potential in ovarian, colorectal, lung, pancreatic, cholangiocarcinoma, and hepatocellular cancers [21]. Ocular toxicities in 26 patients included: photophobia (26.92%; one grade 3), dry eye (23.08%; grades 1-2), ocular pain (15.38%; one grade 3), blurred vision (11.54%; grades 1-2), conjunctivitis (7.69%; grades 1-2), lacrimation (7.69%; grades 1-2), and vitreous floaters (7.69%; grades 1-2) [21]. Limited data warrant further study of toxicity and mechanisms.

*DMUC4064A*:** **DMUC4064A targets MUC16, expressed in most epithelial ovarian cancers (EOCs), and may have anti-EOC activity [22]. It comprises an anti-MUC16 monoclonal antibody and MMAE. Ocular toxicities in 65 patients included: blurred vision (35.38%; two grade 3), dry eye (16.92%; grades 1-2), and keratitis (13.85%; four grade 3) [22]. Further studies are needed to understand these toxicities and their mechanisms.

**Table 5:** Ocular Toxicities Associated with DMUC4064A CTCAE: Common Terminology Criteria for Adverse Events, a standardized classification and severity grading scale developed by the U.S. National Cancer Institute (NCI) for reporting adverse effects in clinical trials and medical practice

Ocular Toxicity	Incidence (n=65)	Percentage	CTCAE Grade(s)
Blurred vision	23	35.38%	1–3 (2 grade 3)
Dry eye	11	16.92%	1–2
Keratitis	9	13.85%	1–3 (4 grade 3)

Drugs for Cancers Associated with Gynecological Cancers

*Gelantamab mafodotin*:** **Belantamab mafodotin is an ADC targeting B-cell maturation antigen, highly expressed on malignant plasma cells in multiple myeloma [26]. Emerging evidence suggests a higher risk of secondary malignancies, including gynecological cancers, with standardized incidence ratios from 1.2 to 1.5 depending on cancer type and treatment [26-28]. It shows significant ocular toxicity, especially keratopathy. In the DREAMM-2 trial, keratopathy was the most common grade 3-4 adverse event: 27% (2.5 mg/kg) and 21% (3.4 mg/kg) [26]. Keratopathy typically appears as microcyst-like epithelial changes and may cause blurred vision or altered visual acuity [27], prompting a Risk Evaluation and Mitigation Strategy and boxed warning [27]. A lyophilised presentation in DREAMM-2 showed similar toxicity: 75% had keratopathy (mostly grade 3-4) and 8% had grade 3-4 blurred vision [28]. In a multicenter study, 68.4% had keratopathy (40.5% grade ≥3) and 36.8% had blurred vision (6.3% grade ≥3) [29]. These findings confirm clinical trial data, showing ocular toxicity is dose-dependent, clinically limiting, and impacts quality of life and adherence. In vivo confocal microscopy reveals early corneal changes, such as sub-basal nerve plexus lesions, which may aid dose adjustment before visible toxicity develops [27].

Strengths and weaknesses

This review provides a comprehensive analysis of the ocular toxicities associated with ADCs used to treat gynecological cancers. It incorporates data from 5,181 patients across six countries, offering a robust dataset for evaluating these adverse effects. The focus on common ocular issues such as blurred vision, keratopathy, and dry eye, along with management strategies, is valuable for clinical practice. However, the review acknowledges certain limitations, such as the lack of comprehensive evidence for some drug combinations (e.g., MIRV with carboplatin), small patient populations, and insufficient long-term follow-up. Inconsistencies in how ocular toxicities were reported and managed across different studies were evident and posed challenges to comparative analysis. To account for this, we qualitatively synthesized ocular toxicity data and categorized findings based on reported severity, intervention strategies, and outcomes. Inconsistent reporting and management across studies underscore the need for future trials to adopt consistent reporting frameworks and standardized techniques, as well as larger trials to validate current findings.

Implications for practice and future research

Although MIRV and TV show promise, particularly for platinum-resistant ovarian cancer and recurrent cervical cancer, their ocular toxicities should be closely monitored. Management strategies, including dose modifications and supportive care, emphasize the importance of vigilant ophthalmological assessment. In general, future research should focus on large-scale, long-term studies that standardize ocular toxicity reporting. Additionally, with the increasing role of artificial intelligence (AI) in cancer diagnostics and treatment monitoring, developing regulatory guidelines and oversight such as FDA approval processes and ethical frameworks are pertinent to protect patient safety and data privacy. Overall, it seems that the integration of AI tools into the clinic may enhance diagnostic accuracy and efficiency. Automated triage systems, second-opinion diagnostic tools, and training aids for in-training radiologists are some examples. While ADCs are a promising frontier in treating gynecological cancers, their associated ocular toxicities require careful monitoring and management.

## Conclusions

This systematic review provides a comprehensive overview of the ocular toxicities associated with ADCs used to treat gynecological cancers. The analysis focuses on MIRV and TV, noting their antitumor activity, especially in platinum-resistant ovarian cancer and recurrent cervical cancer. The evidence shows that MIRV, when given alone or in combination with bevacizumab or carboplatin, provides significant therapeutic benefits but also increases the risk of ocular side effects like blurred vision and keratopathy. TV also shows substantial therapeutic ability to treat a myriad of solid tumors but is associated with conjunctivitis and dry eye among other less frequent ocular issues. There is limited information on trastuzumab duocarmazine, PF-06263507, and DMUC4064A, warranting further research to confirm their antitumor effects and adverse effects. In general, future research should focus on conducting large-scale, long-term studies that standardize the reporting of ocular toxicities associated with ADCs. This will allow clinicians to better understand the mechanisms behind these side effects and facilitate their management. Additionally, standardized protocols for the reporting of ocular toxicities in clinical trials will facilitate better comparisons across studies. In summary, while ADCs are a promising frontier in treating gynecological cancers, their associated ocular toxicities require careful monitoring and management. Additional research may fully characterize these risks, optimizing therapeutic outcomes for patients. 
